# 10-Year Changes in Adiposity in Cameroon School-Age Children: Evidence for Increasing Central Adiposity and Higher Adiposity Levels in Tallest-for-Age Children

**DOI:** 10.1155/2021/6866911

**Published:** 2021-10-15

**Authors:** Lifoter K. Navti, Brice U. S. Foudjo

**Affiliations:** ^1^Department of Biochemistry, The University of Bamenda, P.O. Box 39,Bambili, Bamenda, Cameroon; ^2^Department of Biochemistry, Catholic University of Cameroon (CATUC), Bamenda P.O. Box 782, Bamenda, Cameroon; ^3^Nutrition and Health Research Group (NHRG), Bamenda, Cameroon; ^4^Association Sahelienne de Recherche Appliquee Pour le Developpement Durable (ASRADO), P.O. Box 2449, Djamena, Chad

## Abstract

**Objective:**

To examine changes in measures of adiposity and determine the prevalence of excess adiposity in relation to height in school children between 2010 and 2020.

**Methods:**

5–12-year-old urban school-age children participated in two cross-sectional surveys in 2010 (*n* = 1274) and 2020 (*n* = 1550). Standard procedures were used for anthropometric measurements. Changes in BMI, waist circumference (WC), and waist-to-height ratio (WHtR) and the corresponding proportions of children with excess adiposity were analyzed and adjusted for design variables (class and school type) and age. Children were classified according to quartiles of height z-score and prevalence of excess adiposity estimated across each quartile.

**Results:**

There was a 2.4% and 3.3% increase in adjusted mean BMI and WC, respectively, between 2010 and 2020. The prevalence of central overweight/obesity (WC) and WHtR ≥ 0.5 increased by 7.3% (*X*^2^ = 27.151, *p* < 0.001) and 5.3% (*X*^2^ = 26.117, *p* < 0.001), respectively, between the two surveys except BMI overweight/obesity. The odds of excess adiposity significantly increased in 2020 for central overweight/obesity (WC) (OR 2.8, 95% CI 2.0–3.6) and WHtR ≥ 0.5 (OR 1.8, 95% CI 1.3–2.4) and not for BMI overweight/obesity (OR 1.3, 95% CI 0.8–1.7). The prevalence of BMI overweight/obesity significantly increased from 33% in 2010 to 51.5% in 2020 in the fourth quartile of height z-score (*X*^2^ = 19.198, *p* < 0.001). Similarly, the prevalence of central overweight/obesity (WC) significantly increased from 23.5% in 2010 to 42.4% in 2020 in the fourth quartile of height z-score (*X*^2^ = 18.733, *p* < 0.001).

**Conclusion:**

Central overweight/obesity has increased more than BMI overweight/obesity over the last decade. Children with a higher height-for-age tend to accumulate more adiposity. Objective monitoring of adiposity levels and height of children is needed in future to identify groups for targeted intervention and prevention of chronic diseases.

## 1. Introduction

The increase in the number of overweight/obese children is reaching alarming proportions worldwide and has become an important aspect of global health [1]. Recent evidence from developed countries is showing that the number of children affected with overweight/obesity has plateaued [2]. However, overweight/obesity is increasing at a faster rate in developing countries [3]. This is because the developing countries are undergoing a rapid nutrition transition characterized by increasing income, changes in eating habits, and lifestyle [4]. A recent report indicates that 330 million children between the ages of 5 and 19 years were overweight/obese worldwide in 2016 [5]. Also, a recent review has indicated a transition to higher proportions of overweight/obesity among school-age children in sub-Saharan Africa [6]. The rapid increase is of concern because overweight/obesity in childhood has been shown to be associated with adverse health outcomes including arterial stiffness [7], hypertension [8], type 2 diabetes in adulthood, and coronary artery diseases later in life [9].

Cameroon is a low-income country, and no efforts have been made towards achieving the nutrition targets that concern overweight/obesity, which had been endorsed by the WHO [5]. The distribution of overweight/obesity in the country varies by the administrative region. For instance, recent studies in the Littoral and North West Regions among school-age children have shown prevalence estimates of 14.4% [10] and 17.6% [11], respectively. In the North West Region (NWR), the highest prevalence of overweight/obesity had been recorded amongst the tallest children [11]. Also, a recent nationwide survey indicated that the highest prevalence of overweight/obesity in children is in the Grassfield area of the country that includes the North West Region and West Region [12]. To the best of our knowledge, no study has been carried out in Cameroon to show changes in adiposity levels in school-age children over time.

Many studies that have assessed trends in overweight/obesity have relied on BMI as a measure of adiposity, which has shortcomings. For instance, in monitoring changes in body composition of children over time, it is unclear whether increases in BMI observed are due to increase in lean body mass, increase in fat mass, or both [13]. Also, a previous report indicated that BMI can misclassify a high proportion of children with high body fat because of its low sensitivity [14]. Waist circumference (WC) and waist-to-height ratio (WHtR) are becoming important measures of adiposity in children. There is evidence suggesting that central adiposity (estimated by WC) has a stronger association with adverse health outcomes than adiposity estimated using BMI [15]. Also, WHtR, which is independent of age and gender, has been shown to be more sensitive in predicting risk factors of cardiovascular diseases than BMI [16]. In addition, a study had suggested that WHtR is an easy-to-use measure of central adiposity, and it would prevent the misclassification of many muscular children as overweight/obese that occurs when BMI is used [17]. This study was undertaken to examine recent trends in BMI, WC, and WHtR and determine the prevalence of excess adiposity in relation to stature in two samples of school children between 2010 and 2020 in the NWR of Cameroon.

## 2. Materials and Methods

### 2.1. Study Participants

Data for the current repeated cross-sectional study were obtained from two surveys on school-age children carried out in 2010 and 2020 in the North West Region (NWR) of Cameroon. A total of 2824 (1274 in 2010 and 1550 in 2020) children from three subdivisions in Mezam Division of the NWR between the ages of 5 and 12 years were included in the analysis. The sampling procedure was in two stages: firstly, for each survey, three primary schools were randomly selected in each subdivision from a list of primary schools obtained from the Regional Delegation of Basic Education of the NWR and invited to participate in the study. These primary schools included public, private, and faith-based schools. Secondly, within each school, 40% of children were randomly selected from one class in each target year of study.

**Figure 1:**
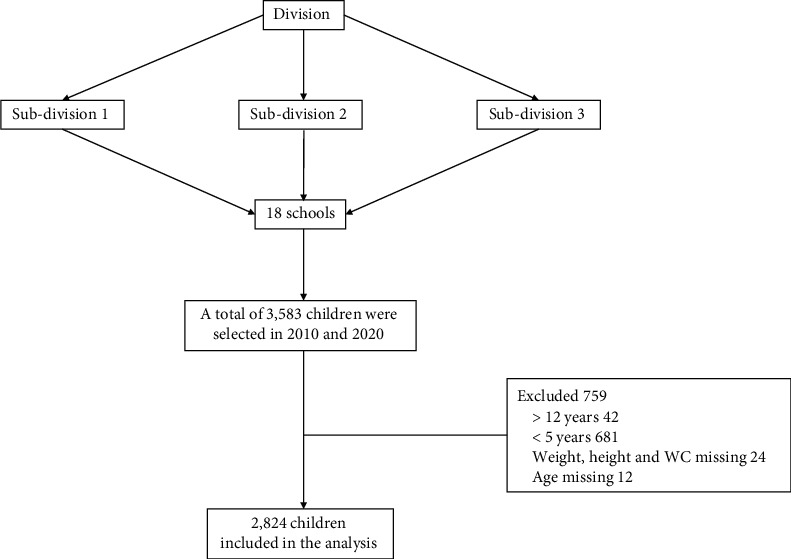
Flowchart of the sampling procedure/cleaning of data.

This study set out to report on changes in the proportions of overweight/obese school-age children between 2010 and 2020. In 2010, the prevalence of overweight/obesity was 16.6%. The sample size was calculated using *p*1=0.166 and *p*2=0.266 in order to be able to detect a 10% difference in the prevalence of overweight/obesity between the two surveys with 80% power and using the statistical significance cutoff of 0.05. The sample size was also adjusted to reflect the inequality of the number of participants in the two surveys [18]. In order to adjust for the correlation of measures of children in the same school, the sample size was inflated. The details of the sample are shown in Figure 1.

### 2.2. Ethical Considerations

Approval to carry out this study was granted by the Institutional Review Board (IRB) of the Catholic University of Cameroon (CATUC), Bamenda (Ref. no. 12/CATUC-IRB/2019). Administrative clearance was obtained from the Regional Delegation of Public Health of the North West Region. Written informed consent and verbal assent were obtained from all head teachers/parents/guardians/and children, respectively, before data collection commenced.

## 3. Data Collection

### 3.1. Anthropometry

Data collection was on school premises during physical education lessons. The same apparatus and the same protocol were followed in the two surveys by well-trained teachers. However, the schools that participated were different in the two surveys.

Height without shoes was measured using a portable stadiometer (Seca 213, Hamburg, Germany) to the nearest 0.1 cm. A digital scale (Omron BF511, Kyoto, Japan) was used to measure body weight to the nearest 0.1 kg. BMI was calculated from the height and weight of the children and recorded in kg/m^2^. Waist circumference was measured as recommended by McCarthy et al. [19] using a nonelastic flexible tape (Seca 201, Germany). The z-scores of height, weight, and BMI were calculated using WHO AnthroPlus, a growth monitoring software for children between the ages of 5 and 19 years, which makes use of the WHO 2007 growth reference data [20]. Waist circumference was also adjusted for age and gender using the McCarthy waist circumference reference data for UK children [19]. Children were classified as overweight/obese (BMI) according to WHO criteria [20]. The 91^st^ centile was used to define central overweight/obesity (WC) according to McCarthy references for WC [19]. WHtR was calculated by dividing the WC (in centimeters) by the height (in centimeters). Based on the fact that excessive upper body fat accumulation poses a health risk, WHtR was used to classify the study participants as “low risk” (<0.5) and “high risk” (≥0.5) [21]. In both surveys, all anthropometric measurements were carried out in duplicates and the average value was calculated and recorded. For reliability analysis, a two-way random-effects model was used to calculate intraclass (intraevaluator) correlation coefficients of anthropometric variables. In 2010, the intraclass correlation coefficients for height, weight, and WC were >0.94, >0.97, and >0.94, respectively. In 2020, the intraclass correlation coefficients were >0.97, >0.96, and >0.96 for height, weight, and waist circumference, respectively.

### 3.2. Statistical Analysis

All statistical procedures were carried out using IBM-SPSS for Windows version 21.0 (IBM Corporation Armonk, NY, USA). The prevalence by survey period was estimated using generalized linear models adjusting for design variables (class and school type) and age. Differences in proportions were compared using the chi-square test. Changes in means of anthropometric variables between the two survey periods were tested using linear regression analysis, with and without adjusting for design variables and age. Odds ratios of the different measures of adiposity were calculated using binary logistic regression analysis adjusting for age and the design variables.

The study participants were then classified into quartiles of height z-score, and prevalence was calculated across each quartile to assess whether the proportion of overweight/obese (BMI) and central overweight/obese (WC) children is increasing with an increase in quartiles of height z-score. The prevalence, mean differences of anthropometric variables, and odds ratios have been presented with their corresponding 95% confidence limits.

Statistical significance was set at *p* < 0.05.

## 4. Results

The descriptive characteristics of the school-age children included in this analysis are presented in Table 1. There were significant (*p* < 0.05) differences in the distribution of participants according to age group, gender, and quartiles of height z-score between the two survey periods. However, a significant difference was not observed when study participants were classified according to school type (*p* > 0.05).

Table 2 shows results of linear regression analysis, which was used to assess changes in means of anthropometric variables between the two surveys without adjusting and after adjusting for design variables and age. Over the 10-year period, the adjusted analysis indicates that the mean BMI and mean WC of the whole sample significantly increased by 2.4% and 3.3%, respectively. On a gender basis, the mean increase in BMI was slightly higher for males (2.9%) than that for females (2.4%). However, over the 10-year period, the increase in mean WC was higher in females (3.4%) than that in males (3.1%). Nevertheless, no significant changes in WHtR were observed between the two surveys. Table 2 also shows that the children were significantly taller and had a higher body weight in 2020 than 2010.

Table 3 shows the prevalence according to BMI, WC, and WHtR for 2010 and 2020. For the whole sample, there was no significant change in the prevalence of BMI overweight/obesity between 2010 and 2020 (*X*^2^ = 0.665, *p*=0.415). However, there was an overall 7.3% increase in the prevalence of central overweight/obesity (WC) between 2010 and 2020, and this was statistically significant (*X*^2^ = 27.151, *p* < 0.001). On a gender basis, the increase in central overweight/obesity (WC) was 7.0% (*X*^2^ = 15.058, *p* < 0.001) and 8.5% (*X*^2^ = 14.962, *p* < 0.001) for males and females, respectively.

Also, there was an overall 5.3% increase in the prevalence of WHtR ≥ 0.5 (“high risk”) between 2010 and 2020 (*X*^2^ = 26.117, *p* < 0.001). In addition, the proportion of those at “high risk” (WHtR ≥ 0.5) increased by 5.6% (*X*^2^ = 16.371, *p* < 0.001) and 5.1% (*X*^2^ = 11.312, *p*=0.002) for males and females, respectively.

**Figure 2:**
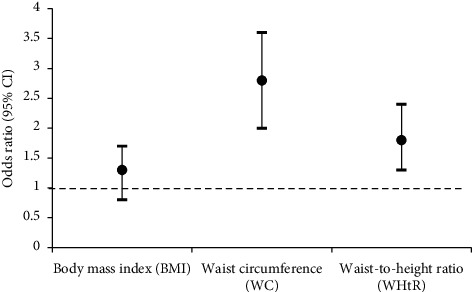
Odds of being BMI overweight/obese, central overweight/obese (WC), and being at “high risk” (WHtR ≥ 0.5) in 2020 when compared to 2010.

Figure 2 shows that the odds of central overweight/obesity (WC) and odds of being at “high risk” (WHtR ≥ 0.5) significantly (*p* < 0.001) increased in 2020 when compared to 2010 as reference. However, the odds of BMI overweight/obesity did not increase significantly.

The odds of having a higher BMI, WC, and WHtR changed over time when analyzed on a gender basis. For instance, in 2020, females had higher odds of being overweight/obese according to BMI [OR 2.36, CI (1.65–3.38), *p* < 0.001] and WC [OR 2.89, CI (1.83–5.51), *p* < 0.001] when compared to 2010. However, the odds of being at “high risk” according to WHtR was not significant for females [OR 1.94, CI (0.96–3.94) *p*=0.066]. Also, in 2020, males had higher odds of being overweight/obese according to BMI [OR 2.59, CI (1.78–3.76), *p* < 0.001] and being at “high risk” according to WHtR [OR 3.79, CI (1.88–8.75), *p*=0.001] when compared to 2010. However, the odds of being centrally overweight/obese according to WC was not significant for males [OR 1.41, CI (0.94–2.11), *p*=0.094].

When the study participants were classified according to quartiles of height z-score, the mean height in 2010 for the first, second, third, and fourth quartiles of height z-score was 124.7 cm, 131.0 cm, 133.4 cm, and 137.1 cm, respectively. In 2020, the mean height for the first, second, third, and fourth quartiles of height z-score was 126.7 cm, 131.1 cm, 137.4 cm, and 144.0 cm, respectively.

**Figure 3:**
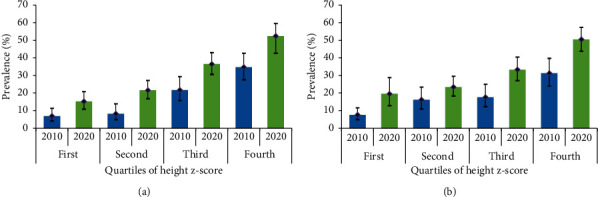
Adjusted prevalence (%, 95% CI) of BMI overweight/obesity across quartiles of height z-score in males (a) and females (b).

Figure 3 shows the changes in the prevalence of BMI overweight/obesity in relation to height in males and females. For both surveys, the prevalence of BMI overweight/obesity increased with increasing quartiles of height z-score. In 2020, the prevalence of BMI overweight/obesity was higher across all quartiles of height z-score than 2010. However, a significant difference in the prevalence of BMI overweight/obesity was observed between the two surveys only in the fourth quartile of height z-score in females (*X*^2^ = 11.838, *p*=0.001). This was not significant for males (*X*^2^ = 0.143, *p*=0.391).

**Figure 4:**
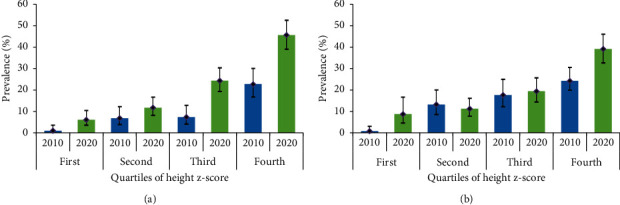
Adjusted prevalence (%, 95% CI) of central overweight/obesity across quartiles of height z-score in males (a) and females (b).

Figure 4 also shows that the prevalence of central overweight/obesity (WC) increased with increasing quartiles of height z-score in both surveys for males and females. There were significant differences in the prevalence of central overweight/obesity (WC) between 2010 and 2020 in both males (*X*^2^ = 11.429, *p*=0.001) and females (*X*^2^ = 7.817, *p*=0.005) in the fourth quartile of height z-score.

## 5. Discussion

This analysis set out to examine changes in measures of adiposity (BMI, WC, and WHtR) and also determine the prevalence of excess adiposity in relation to height over a 10-year period in two groups of children in the North West Region of Cameroon.

This study confirms that there is no change in the adjusted prevalence of BMI overweight/obesity between the two surveys. However, the data show an increasing trend in both central overweight/obesity (WC) and WHtR ≥ 0.5 among all children. This means central overweight/obesity is increasing more than BMI overweight/obesity. A previous study in Norway had shown an increase in trends of WC but not BMI over a 6-year period after adjusting for school type, tanner stage, and age [22]. Several studies in different countries have shown that the central obesity assessed using WC in children has been increasing over time. For instance, reports by Liang et al. [23], Griffiths et al. [24], and Suder et al. [25] showed that WC increased at a relatively faster rate than BMI among Chinese, British, and Polish children, respectively. Also, central obesity assessed using central skinfold thickness was found to increase at a faster rate than BMI in Brazilian children [26]. In addition, a study by Freedman et al. [27] indicated that secular increases in WC observed in US children from 1998–1994 through 2011-2012 are independent of changes in BMI. As observed in this study, other authors had also indicated that this increase in WC was more in girls than in boys [24, 26]. However, a study in Brazilian children reported a higher increase in central obesity in boys than girls over a five-year period [28]. The increase in waist circumference observed in this study could be due to a transition to unhealthy eating habits, increase in sedentary lifestyle, and physical inactivity among children in sub-Saharan Africa [4]. In fact, a previous study in the NWR of Cameroon had indicated that an increase in sedentary lifestyle (>3 hours/day) was associated with a 1.37 mm increase in triceps skinfold thickness [29]. The prevalence of WHtR ≥ 0.05 in our study also increased in both males and females during the 10-year period. This is similar to a previous study, which indicated that WHtR increased over time in three different samples of children, especially in girls [24]. Our analysis indicated that there is no significant change in the prevalence of BMI overweight/obesity over time. This is contrary to a report by Lazzeri et al. [30], which indicated that BMI overweight/obesity decreased over a ten-year period in Tuscan school children. The authors highlighted different initiatives that could have contributed to the decrease including official dietary guidelines, nutrition recommendations, national surveillance, and programmes aimed at encouraging fruit and vegetable intake in children. Cameroon is yet to attain the WHO nutrition targets that concern overweight/obesity [5], and our analysis has confirmed that the children are now having a more centralized distribution of body fat.

Logistic regression indicated gender disparities in the odds of being overweight/obese (BMI), central overweight/obese (WC), and being “at risk” (WHtR ≥ 0.5) over time. However, when the whole sample was taken into consideration, the odds of central overweight/obesity (WC) and WHtR ≥ 0.5 significantly increased over time. However, the increase in odds of BMI overweight/obesity was not significant. This increase in risk of central overweight/obesity in our sample is concerning. This is because evidence indicates that children with central overweight/obesity are at risk of developing adverse health outcomes early in life including cardiovascular disease and type 2 diabetes [9]. It is important to point out that the authors of the abovementioned studies took WC measurements at different anatomical sites, which could account for the differences observed. However, a previous study in children had shown that the relationships between WC (measured at four different anatomical sites) and cardiometabolic risk factors were similar within race and gender groups [31].

This analysis has confirmed that the prevalence of BMI overweight/obesity and central overweight/obesity (WC) increased with increasing quartiles of height z-score in the two surveys. The highest prevalence was recorded amongst the tallest-for-age children. Also, the prevalence of BMI overweight/obesity and central overweight/obesity was higher in 2020 than 2010 within each quartile of height z-score.

A previous analysis in the NW Region of Cameroon had shown that children who have a higher height-for-age accumulated more adiposity [11]. Our current analysis indicates that this association has persisted over time when BMI and WC are used to assess adiposity suggesting that tallness could predict both the level and distribution of adiposity in children. Previous evidence from other countries has shown that taller children tend to be overweight/obese [32–34]. Also, a report had shown higher adiposity levels amongst the tallest UK children from different age groups [35]. However, the BIA equipment used in the study had been shown to underestimate adiposity in children.

The height-adiposity relationship observed in this study could be explained from a nutrition perspective. There is evidence suggesting that high protein intake early in life is associated with increased risk of obesity [36], and the high protein intake has been suggested to contribute to an imbalance in some hormonal levels in obese children. For instance, a previous report indicated that obese children have higher levels of insulin-like growth factor 1 (IGF-1) and lower levels of growth hormone (GH) [37], all of which result from high protein intake [38]. The high levels of IGF-1 promote adipose tissue hyperplasia and growth [39]. Also, the deceased levels of GH result in a decrease in its lipolytic effect [37], which results in an increase in adiposity levels.

However, the source of protein and the timing need to be considered because a study had indicated that diary protein at the age of 1 year is associated with higher levels of adiposity at age 7 years [38]. Also, a report had suggested that this relationship between height and adiposity could result from an accumulation of body fat at levels higher than that required for linear growth [33]. In addition, previous reports had indicated that taller children were fatter and more insulin resistant [40, 41], suggesting that insulin resistance could explain to some extent the higher levels of adiposity observed among the tallest children in our study. However, this needs further investigation.

This study has limitations worth mentioning. Our data are from two different cross-sectional surveys and can be used neither to establish causality nor in the prediction of future trends. Also, in the absence of reference data for WC in our setting, we used the UK growth reference data for WC in our analysis to define central overweight/obesity, which may not be reflective of Cameroonian children. In addition, we could not adjust for other important variables that could influence adiposity such as puberty and dietary habits. Thus, we do not have evidence to indicate whether our current observations are as much a result of a high protein intake in our sample.

The current study had strong points worth mentioning. Our data have provided a glimpse of trends over the last decade for the first time in Cameroon school-age children. Also, other objective measures of adiposity (WC and WHtR) were included, which could be used alongside BMI. In addition, we included design/socioeconomic variables (class and school type) in our analysis, and this avoided an overestimation of the differences observed between the two surveys.

## 6. Conclusions

To conclude, the proportion of children with central overweight/obesity has significantly increased. The odds of central overweight/obesity (WC), and being “at risk” according to WHtR, has increased over ten years, and this is concerning. Our study provides baseline data for future comparisons. We propose the establishment of a surveillance system and school-based preventive measures for children. We also recommend the use of waist circumference as an additional tool for health promotion and early diagnosis of central overweight/obesity. The prevalence of BMI overweight/obesity and central overweight/obesity (WC) was the highest amongst the tallest children, and this association has persisted over time. Thus, we are suggesting that standing height could be used in the clinical setting to predict adiposity. However, longitudinal studies will be needed in the future to ascertain our claim. Also, future studies are needed to identify the different biological, environmental, and behavioral drivers of adiposity in order to reduce the risk of chronic diseases.

## Figures and Tables

**Table 1:** Descriptive characteristics of the study population.

Variable	2010		2020		*p* value
	*N* = 1274		*N* = 1550		
	*n*	%	*n*	%	
*Age group (years)*					<0.001
5-6	266	20.8	448	29.0	
7-8	376	29.5	442	28.5	
9-10	488	38.3	392	25.3	
11-12	146	11.4	266	17.2	

*Gender*					0.006
Female	638	50.1	694	44.8	
Male	636	49.9	856	55.2	

*School type*					0.182
Public	642	50.4	742	47.9	
Private/faith-based	632	49.6	808	52.1	

*Quartiles of height SDS*					<0.001
First quartile	440	34.5	290	18.7	
Second quartile	282	22.1	436	28.1	
Third quartile	274	21.5	416	26.8	
Fourth quartile	278	21.8	408	26.3	

**Table 2:** Means of the anthropometric parameters of study participants between 2010 and 2020.

Variables	Unadjusted analysis							Adjusted analysis ^*∗*^		
	2010		2020		Mean difference	95% CI	*p* value	Mean difference	95% CI	*p* value
	Mean	95% CI	Mean	95% CI						
*Whole sample*										
BMI (kg/m^2^)	17.0	(16.8–17.2)	17.3	(17.1–17.5)	0.3	(−0.0–0.5)	0.054	0.4	(0.2–0.7)	0.001
BMI z-score	0.19	(0.12–0.27)	0.32	(0.23–0.41)	0.13	(0.00–0.25)	0.042	0.15	(0.03–0.27)	0.017
WC (cm)	58.0	(57.6–58.5)	59.5	(58.9–60.1)	1.5	(0.7–2.2)	<0.001	1.9	(1.3–2.6)	<0.001
WC z-score	0.34	(0.28–0.41)	0.54	(0.47–0.62)	0.20	(0.09–0.30)	<0.001	0.20	(0.10–0.31)	<0.001
WHtR	0.442	(0.440–0.445)	0.441	(0.438–0.445)	−0.001	(−0.006–0.003)	0.574	−0.003	(−0.007–0.002)	0.242
Height (cm)	131.5	(130.6–132.5)	135.3	(134.2–136.4)	3.8	(2.3–5.2)	<0.001	5.2	(4.5–6.0)	<0.001
Height z-score	−0.12	(−0.22–−0.01)	0.83	(0.76–0.90)	0.95	(0.83–1.07)	<0.001	0.94	(0.82–1.06)	<0.001
Weight (kg)	30.0	(29.3–30.7)	32.6	(31.8–33.4)	2.6	(1.5–3.7)	<0.001	3.6	(2.9–4.2)	<0.001
Weight z-score	−0.03	(−0.1–0.03)	0.69	(0.61–0.77)	0.72	(0.61–0.82)	<0.001	0.73	(0.62–0.84)	<0.001

*Females*										
BMI (kg/m^2^)	16.9	(16.7–17.2)	17.2	(16.9–17.4)	0.2	(−0.1–0.6)	0.242	0.4	(0.1–0.7)	0.026
BMI z-score	0.28	(0.18–0.38)	0.27	(0.15–0.39)	−0.01	(−0.18–0.16)	0.905	0.02	(−0.15–0.18)	0.837
WC (cm)	58.2	(57.5–58.8)	59.6	(58.8–60.5)	1.4	(0.3–2.5)	0.012	2.0	(1.0–3.0)	<0.001
WC z-score	0.31	(0.22–0.41)	0.54	(0.44–0.66)	0.24	(0.09–0.38)	0.002	0.24	(0.09–0.38)	0.002
WHtR	0.443	(0.439–0.446)	0.444	(0.439–0.449)	0.001	(−0.006–0.008)	0.753	−0.001	(−0.007–0.006)	0.859
Height (cm)	131.8	(130.5–133.2)	134.6	(133.3–136.1)	2.8	(0.8–4.9)	0.006	4.7	(3.7–5.6)	<0.001
Height z-score	−0.12	(−0.26–0.02)	0.76	(0.66–0.85)	0.88	(0.71–1.04)	<0.001	0.87	(0.71–1.03)	<0.001
Weight (kg)	30.0	(29.1–30.9)	32.1	(31.0–33.2)	2.1	(0.6–3.5)	0.005	3.2	(2.3–4.2)	<0.001
Weight z-score	−0.07	(−0.10–−0.03)	0.62	(0.51–0.72)	0.69	(0.56–0.82)	<0.001	0.70	(0.57–0.83)	<0.001

*Males*										
BMI (kg/m^2^)	17.0	(16.8–17.3)	17.4	(17.1–17.7)	0.3	(−0.07–0.76)	0.104	0.5	(0.1–0.8)	<0.018
BMI z-score	0.17	(0.10–0.23)	0.30	(0.25–0.44)	0.13	(0.07–0.27)	0.003	0.14	(0.08–0.29)	0.001
WC (cm)	57.9	(57.2–58.5)	59.4	(58.6–60.1)	1.5	(0.5–2.5)	0.004	1.8	(1.0–2.6)	<0.001
WC z-score	0.37	(0.27–0.48)	0.54	(0.42–0.65)	0.16	(0.01–0.31)	0.040	0.17	(0.02–0.32)	0.032
WHtR	0.442	(0.438–0.446)	0.438	(0.433–0.442)	−0.004	(−0.010–0.001)	0.139	−0.005	(−0.011–0.000)	0.063
Height (cm)	131.2	(129.9–132.5)	136.1	(134.5–137.7)	4.9	(2.8–6.9)	<0.001	5.9	(4.9–7.0)	<0.001
Height z-score	−0.11	(−0.26–0.03)	0.92	(0.82–1.02)	1.04	(−1.43–−0.87)	<0.001	1.03	(0.85–1.21)	<0.001
Weight (kg)	30.0	(29.0–30.9)	33.2	(32.0–34.4)	3.2	(1.7–4.8)	<0.001	4.0	(2.9–5.0)	<0.001
Weight z-score	0.00	(−0.12–0.13)	0.77	(0.65–0.88)	0.77	(0.60–0.94)	<0.001	0.77	(0.60–0.94)	<0.001

CI; confidence interval,  ^*∗*^adjusted for age, class, and school type.

**Table 3:** Prevalence of BMI overweight/obesity, central overweight/obesity, and WHtR ≥ 0.5 over time.

	Status	2010				2020			
		Males (*N* = 636)		Females (*N* = 638)		Males (*N* = 856)		Females (*N* = 694)	
		%	(95% CI)	%	(95% CI)	%	(95% CI)	%	(95% CI)
BMI z-score	Overweight	15.1	(12.5–18.1)	13.2	(10.8–16.0)	15.4	(13.2–17.9)	14.1	(11.7–16.9)
	Obesity	1.9	(1.1–3.3)	3.1	(2.0–4.8)	2.8	(1.9–4.1)	3.2	(2.1–4.8)

WC z-score	Overweight	8.5	(6.6–10.9)	12.2	(9.9–15.0)	11.9	(9.9–14.3)	15.9	(13.3–18.8)
	Central obesity	0.9	(0.4–2.0)	3.8	(2.5–5.5)	4.4	(3.3–6.0)	8.6	(6.8–10.9)

WHtR	High risk	4.7	(3.3–6.7)	5.6	(4.1–7.7)	10.3	(8.4–12.5)	10.7	(8.6–13.2)

CI; confidence interval.

## Data Availability

The data used to support the findings of this study are available from the corresponding author upon reasonable request.
